# Histone demethylase JARID1B/KDM5B promotes aggressiveness of non-small cell lung cancer and serves as a good prognostic predictor

**DOI:** 10.1186/s13148-018-0533-9

**Published:** 2018-08-09

**Authors:** Kuang-Tai Kuo, Wen-Chien Huang, Oluwaseun Adebayo Bamodu, Wei-Hwa Lee, Chun-Hua Wang, M. Hsiao, Liang-Shun Wang, Chi-Tai Yeh

**Affiliations:** 1 Division of Thoracic Surgery, Department of Surgery, Shuang Ho Hospital, Taipei Medical University, New Taipei City, Taiwan; 2 Division of Thoracic Surgery, Department of Surgery, School of Medicine, College of Medicine, Taipei Medical University, Taipei, Taiwan; 3 Division of Thoracic Surgery, Department of Surgery, MacKay Memorial Hospital, Taipei, Taiwan; 4 MacKay Medical College, Taipei, Taiwan; 5 Department of Medical Research and Education, Shuang Ho Hospital, Taipei Medical University, New Taipei City, Taiwan; 6 Division of Hematology/Oncology, Department of Medicine, Shuang Ho Hospital, Taipei Medical University, New Taipei City, Taiwan; 7 Department of Pathology, Shuang Ho Hospital, Taipei Medical University, New Taipei City, Taiwan; 8 Department of Dermatology, Taipei Tzu Chi Hospital, Buddhist Tzu Chi Medical Foundation, New Taipei City, Taiwan; 9 School of Medicine, Buddhist Tzu Chi University, Hualien, Taiwan; 10 Genomics Research Center, Academia Sinica, Taipei, Taiwan

**Keywords:** JARID1B, Lung cancer, Prognosis, Cancer stem cells, c-Met

## Abstract

**Background:**

Lung cancer is the leading cause of cancer death worldwide. Recently, epigenetic dysregulation has been known to promote tumor progression and therefore may be a therapeutic target for anticancer therapy. JARID1B, a member of histone demethylases, has been found to be related to tumorigenesis in certain kinds of cancers. However, its biological roles in non-small cell lung cancer (NSCLC) remain largely unclear.

**Methods:**

We firstly examined the expression of JARID1B in surgical specimens and six NSCLC cell lines. Then, we evaluated the relationship between JARID1B expression and clinicopathologic parameters in 72 NSCLC patients, thereby established its prognostic importance. We subsequently studied the functional roles of JARID1B in tumorigenesis to verify its clinicopathologic significance.

**Results:**

Our results showed that JARID1B was overexpressed in NSCLC cells and JARID1B overexpression was associated with tumor size, lymph node metastasis, advanced stages, and poor overall survival in NSCLC patients. JARID1B overexpression resulted in increased cell proliferation and formation of tumorspheres and correlated positively with the expression of cancer stem cells (CSCs) and epithelial-mesenchymal transition (EMT) markers, while the c-Met signaling pathway was actively involved. It also correlated with the strength of resistance to cisplatin and doxorubicin. On the contrary, downregulation of JARID1B expression by applying shRNA or JARID1B inhibitor PBIT reversed these phenomena.

**Conclusions:**

JARID1B worsens prognosis of NSCLC patients by promotion of tumor aggressiveness through multiple biological facets which were associated with activation of the c-Met signaling, and can be a novel prognostic biomarker and therapeutic target for NSCLC.

**Electronic supplementary material:**

The online version of this article (10.1186/s13148-018-0533-9) contains supplementary material, which is available to authorized users.

## Background

Lung cancer is one of the most frequently diagnosed malignancies and the leading cause of cancer death worldwide, with an estimated global incidence of 1.82 million and mortality of 1.59 million in 2012 [1]. Non-small cell lung carcinoma (NSCLC) accounts for 85% of all lung cancers, and only few patients are diagnosed with early disease [2]. Despite the advance of treatment in all aspects, the all-stage 5-year survival of lung cancer remains less than 20% [3]. During the past decade, new chemotherapeutic agents and target therapies have improved survival of lung cancer patients but the effect was not overwhelming, mostly due to development of therapeutic resistance after treatment [4, 5]. Several studies have shown a small subpopulation of tumor cells called cancer stem cells (CSCs) modulate tumor initiation, growth, metastasis, and resistance to anticancer therapy [6, 7]. These CSCs are characterized by enhanced propensity for self-renewal, unrestricted proliferation, de-differentiation, and tumor propagation. Several somatic stem cell markers including SOX2, KLF4, c-Myc, NANOG, and OCT 3/4 have been applied to identify CSCs, and some additional markers such as ALDH and CD133 have also been proposed for lung cancer stem cells (LCSCs) [8].

The last decade has witnessed increasing implication of enhanced mesenchymal-to-epithelial transition (MET) signaling in the formation, resistance to therapy, and progression of NSCLC [9]. It has also been suggested that MET is required for epithelial growth factor (EGF)-induced cell invasion and motility in EGFR wild-type NSCLC cells, especially as the pharmacological inhibition of c-Met or its siRNA knockdown reduced EGF-induced invasion and motility, indicating that EGFR requires c-Met activity and/or expression to maximize the invasive phenotypes of NSCLC cells [10]. However, the molecular mechanisms of these c-Met activities in NSCLC cells remain unclear, especially its epigenetic underlying molecular mechanism, thus forming a basis for continued exploration of the pharmacologic and molecular targetability, as well as the epigenetic modulation of MET signaling in NSCLC patients.

There is accumulating evidence that besides the genetic mutations, epigenetic changes and chromatin dynamics are actively involved in the initiation and disease progression of several malignancies, including NSCLC [11–14]. However, the underlying mechanisms of epigenetic activities such as histone modification in NSCLC tumorigenesis have largely been underexplored until now. Like other types of histone modification, methylation and demethylation of the lysine residue of the histone protein modulate genetic activities while concurrently serve as a transcription switch of gene expression in both physiological and diseased condition. In the past decade, several studies have demonstrated the oncogenicity of histone demethylases and implicated their dysregulation in tumor formation and progression [15–17].

JARID1B, also known as KDM5B or PLU-1, is one member of the Jumonji, AT-rich interactive domain 1 (JARID1) histone demethylase protein family that possesses H3K4 histone demethylase activity [18, 19]. Our previous studies have shown that JARID1B was associated with tumorigenicity in oral cancer and breast cancer [20, 21]. Recently, it was also reported that an inhibitor of KDM5 family could reduce survival of drug-tolerant cancer cells, including NSCLC cells [22]. This implied the possible link between JARID1B and LCSCs. Nevertheless, the functional and prognostic roles of JARID1B in NSCLC have not been well clarified so far. Meanwhile, the relationship between JARID1B, LCSCs, and c-Met is also obscure. In this study, we evaluated the expression of JARID1B in NSCLC tumor tissues and cell lines, analyzed its clinicopathologic significance in NSCLC patients, and finally investigated its biological roles in NSCLC tumorigenesis.

## Methods

### Cell lines and culture

The human bronchial epithelial cell line BEAS-2B and NSCLC cell lines, CL1-0, CL1-5, A549, and PC9 were grown in Dulbecco’s modified Eagle medium (Gibco®DMEM, Thermo Fisher Scientific Inc., Waltham, MA, USA), while NSCLC cell lines H441 and H1299 were grown in Gibco®RPMI1640 medium (Thermo Fisher Scientific Inc.). The culture media contain 10% FBS (Gibco, Thermo Fisher Scientific Inc.) supplemented with penicillin (100 U/mL) and streptomycin (100 mg/mL) (Gibco, Thermo Fisher Scientific Inc.). Cells were incubated at 37 °C in a 5% CO_2_ humidified atmosphere. Culture media were changed every 72 h and cells passaged at 80% confluence. All cell lines were purchased from ATCC.

### Western blot analysis

Normal bronchial epithelial and NSCLC cells were lysed in RIPA lysis buffer; total protein were quantified by BCA protein assay kit (Thermo Fisher Scientific Inc.) and then analyzed by Western blot assay. Primary antibodies used were listed in Additional file 1: Table S1. Secondary antibodies were Alexa Fluor 680-conjugated affinity purified anti-mouse or anti-rabbit IgG (Invitrogen, Thermo Fisher Scientific Inc.) detected using the UVP Imaging.

### Immunohistochemical staining and scoring

This study was conducted in a cohort of patients with lung cancer who underwent resection. at Taipei Medical University Shuang-Ho hospital, Taipei, Taiwan, between January 2010 to December 2017. A predesigned data collection format was used to review the patients’ medical records for evaluation of clinicopathologic characteristics and survival outcomes. The study was reviewed and approved by the institute review board (IRB:201403007). Clinical samples from NSCLC patients were fixed in 10% formalin, embedded in paraffin, deparaffinized, and then rehydrated. For immunohistochemical (IHC) staining, rehydrated sections were subjected to antigen retrieval and their endogenous peroxidase activity blocked for 30 min in 1% H_2_O_2_/PBS solution. After blocking, the slides were exposed to JARID1B antibody (1:200), c-Met (1:150), or Vimentin (1:200) at 4 °C overnight, at 4 °C overnight, washed and incubated in biotinylated link universal antiserum for 1 h at room temperature. Slides were then rinsed, and stain was developed using the chromogen, 3, 3-diaminobenzidine hydrochloride. Finally, sections were rinsed with ddH_2_O and counterstained with hematoxylin. Slides were observed under microscope, with the selection of five fields of view randomly. Evaluation and quantification of JARID1B, c-Met, or Vimentin expression were done manually by two independent investigators in a blind manner. The percentage of stained area to the selected field was recorded in a 5% interval, ranging from 0 to 100%. The staining intensity was graded into three categories (absent or weak, 1; moderate, 2; strong, 3). Quick score (Q-score) was derived from the product of percentage (*P*) of tumor cells with characteristic IHC staining (0–100%) and the intensity (*I*) of IHC staining (1–3) (*Q*  = *P* × *I*; maximum = 300). For survival analysis, we used Q-score = 150 as a cutoff value to divide the patients into two groups.

### JARID1B-knockdown cell lines

NSCLC H1299 and H441 cells were infected with JARID1B small hairpin RNA (shRNA, Clone ID: TRCN0000329952, target sequence: ATCGCTTGCTTCATCGATATT for shJARID1B-1 and GTGCCTGTTTACCGAACTAAT for shJARID1B-2), or vector (pLKO_TRC005) obtained from the National RNAi Core Facility, Academia Sinica, Taiwan. Treatment with puromycin did selection of positive shJARID1B-1 and shJARID1B-2. Both H1299 and H441 cells were used for phenotypic assays, but only H441 cell lysates were prepared 48 h after transfection and used for Western blot or cytotoxicity assays.

### Sulforhodamine B (SRB) cell proliferation assay

The sulforhodamine B (SRB) assay is used for cell density determination, and the principle has been well described [23]. Cells with the amount of 5 × 10^3^ cells/well from H1299 and H441 were seeded into a 96-well plate and allowed to grow and attach in 200 μL serum-free RPMI 1640 for 24 h. After cell attachment, the media were changed to new serum-free RPMI 1640 and incubated for 24, 48, and 72 h, respectively. At the end of incubation, the cells were washed with PBS for three times. Then, 100 μL of SRB (Sigma-Aldrich, St. Louis, MO) solution 0.4% (*w*/*v*) in 1% acetic acid was added to each well and incubated at room temperature for 1 h. After staining, unbound dye was removed and bound stain was solubilized with 200 μL/well 10 mM Tris base for 30 min. The absorbance was read on an automated microplate reader (96 well) at 490 nm. Each condition of each cell line was repeated for six times.

### Colony formation assay

Five thousand wild-type or JARID1B shRNA H441 cells/60-mm dishes were seeded and cultured in complete growth medium with 0.125 μg/mL puromycin (Gibco, Thermo Fisher Scientific Inc.) for 10–12 days. Colonies were then fixed in ice-cold methanol and stained with 0.1% crystal violet solutions, photographed, and visible colonies counted under microscope. The assays were performed in triplicate.

### Flow-cytometric analysis of cell cycle progression

Cell cycle progression was analyzed by resuspension of 1 × 10^6^ H441 WT or JARID1B shRNA H441 cells per 1 mL PBS, followed by addition of 0.05 mg/mL propidium iodide (PI) in a 0.1% Triton X-100/0.1% sodium citrate solution. Cells were harvested, washed with cold PBS twice, and fixed in 70% ethanol at − 20 °C overnight. The cells were then centrifuged (1500 rpm, 10 min) and washed twice using phosphate-buffered saline (PBS). Next, the cells were resuspended in 0.5 mL of PBS containing 50 μg/mL RNase A for 1 h at 37 °C. The cells were then loaded with 65 μg/mL PI for 30 min in the dark at 4 °C. The percentage of cells in distinct phases of the cell cycle was measured by flow cytometry (FACSCalibur, BD Biosciences) with excitation set at 488 nm and emission detected at the FL-2 channel (565–610 nm). The assays were performed in triplicate.

### Cell migration and invasion assay*s*

NSCLC cells were cultured to 90% confluence in 6-well plates, and then, a scratch was made horizontally through the confluent cells using sterile 10-μL pipette tips. Phosphate-buffered saline (PBS) was used to wash off displaced cells and cellular debris. Five visual fields were randomly selected in each dish for comparison of wound closure. For assessment of cell migration, images were captured under microscope at 0 and 24 h. For invasion assay, matrigel-coated transwell inserts with micropore membranes (BD Biosciences, San Jose, CA, USA) were placed in 24-well plates. 3 × 10^4^ cells were plated in 100 μl of medium containing 1% FBS in the upper chamber, while the lower chamber was filled with 600 μl complete growth medium. Cells were incubated in 5% CO_2_ humidified atmosphere at 37 °C for 48 h. The non-invading cells were scraped from the upper chamber of each insert with cotton swab, and invaded cells attached to the lower surface of the insert membrane were incubated in 0.1% crystal violet at 37 °C for 30 min, washed twice with PBS, and viewed under a microscope.

### Tumorsphere formation assay

For the analysis of sphere forming ability, H441 NSCLC cells were cultured under serum-deprived conditions and in Ultra-Low Attachment Plates (Corning Incorporated). H441 cells (10^3^ cells/mL) were suspended and seeded in the tumorsphere medium containing 20 ng/mL epidermal growth factor, 10 ng/mL basic fibroblast growth factor, 5 μg/mL insulin, 0.4% bovine serum albumin. Approximately 3–5 days of incubation, tumorsphere numbers were counted under a phase-contrast microscope using the × 40 magnification lens. The ability of tumor formation was represented by the average number of spheres obtaining from counts from different views (at least three random fields).

### Immunofluorescence staining

H441 sphere cells were seeded on 24-well plates with a coverslip on the bottom of each well. After shJARID1B treatment, cells were washed twice with PBS, fixed with 4% formaldehyde, and probed with primary antibody (JARID1B, and SOX-2) at 4 °C for overnight. Fluorophore-conjugated antibody (Alexa Fluor; Life Technologies) was used to track the in-situ interaction of protein-primary antibody. Double-stranded DNA staining (4′,6-diamidino-2-phenylindole (DAPI); Invitrogen, USA) was used as nuclear staining. The fluorescence signal was captured under confocal microscopy (Nikon, Japan).

### Isolation of side-population (SP) cancer cells using fluorescence-activated cell sorting (FACS)

H441 WT or JARID1B shRNA H441 cells in logarithmic growth phase were harvested using trypsin/EDTA and resuspended in pre-warmed appropriate culture media (according to each cell type), at a concentration of 5 × 10^5^ viable cells/mL. Single-cell suspensions were incubated with Hoechst-33342 dye (2.5 μg/mL, Sigma) at 37 °C for 90 min in a water bath and in the dark, with occasional agitation to prevent cell aggregation. Negative control samples were treated with Verapamil (50 μM, Sigma), a wide spectrum ATP-binding cassette (ABC) transporter inhibitor for 15 min, before being incubated with Hoechst-33342. Afterwards, cells were washed with ice-cold PBS, centrifuged at 4 °C, and resuspended in ice-cold PBS with propidium iodide (1 μM), to identify and exclude dead cells. All samples were maintained at 4 °C until flow cytometry acquisition. Hoechst-33342 dye was excited at 355 nm using a UV laser, and its dual wavelengths were detected using 450/50-nm band-pass and 450LP filters (Hoechst-33342 Blue), and 655LP filter (Hoechst-33,342 Red), for the discrimination of side-population cells. At least 10,000 events were acquired in the side-population region. Dead cells were excluded by gating propidium iodide-positive cells on forward vs. side scatter dotplots. Data were acquired using FACSAria™ III sorter (BD Biosciences, Taiwan).

### Statistical analysis

All assays were performed in triplicate. Reported data results are expressed as means ± S.E.M. All statistical analyses were performed using GraphPad prism (v.6.0. GraphPad Software Inc., CA, USA). Survival analysis was performed using the Kaplan-Meier plots and log-rank test. The correlation between JARID1B expression and the clinicopathologic parameters was assessed by the *χ*^2^ test and bivariate analysis. For comparisons between two groups, student’s *t* test was used while for more than two groups, one-way ANOVA was used. A *p* value < 0.05 was considered statistically significant (*p < 0.05, **p < 0.01, ***p < 0.001).

## Results

### JARID1B is overexpressed in NSCLC tissues and cell lines

**Fig. 1:**
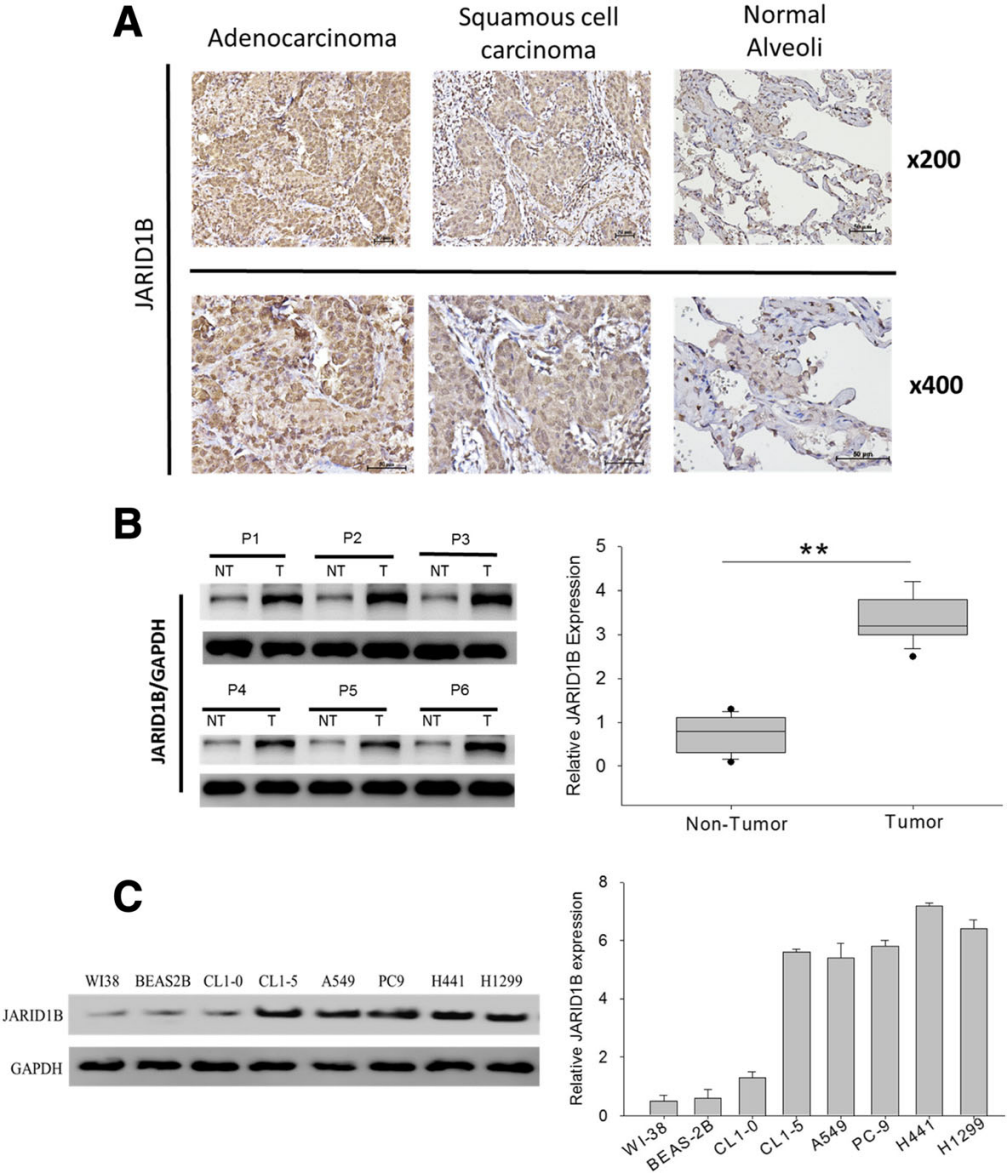
JARID1B is overexpressed in NSCLC tissues and cell lines. **a** IHC staining of clinical specimens. JARID1B overexpression was demonstrated by strong staining intensity of NSCLC tissues while scanty staining was observed in normal alveoli tissues. **b** Representative Western blot data showed upregulation of JARID1B in most of the NSCLC tumor tissues (T) as compared to the adjacent non-tumor (NT) lung tissues. **c** Western blot showed elevated expression of JARID1B in NSCLC cell lines but very weak expression in the human alveolar epithelial cell line BEAS-2B. The H441 and H1299 cells had stronger JARID1B expression. GAPDH served as the loading control

Nuclear staining of JARID1B was intense, but cytoplasmic staining to some extent was also noted. We demonstrated that while the normal alveoli tissues showed scanty JARID1B staining, JARID1B expression was obviously stronger in the NSCLC tissues (Fig. 1a). To further verify this finding, we comparatively evaluated the expression of JARID1B in paired tumor and adjacent non-tumor tissues by Western blot and observed that JARID1B was overexpressed in the tumor (T) samples as compared to the adjacent non-tumor (NT) samples (Fig. 1b). We subsequently analyzed the expression of JARID1B in six widely used NSCLC cell lines, CL1-0, CL1-5, A549, PC9, H441, and H1299 as well as in the human fibroblast cell line WI-38 plus human alveolar epithelial cell line BEAS-2B. The results showed that JARID1B expression was elevated in almost all the NSCLC cell lines as compared to WI-38 or BEAS-2B. JARID1B expression was stronger in H1299 and H441 cells; modest in CL1-5, A549, and PC9 cells; and mild in CL1-0 cells (Fig. 1c). Notably, the more aggressive CL1-5 cells had stronger JARID1B expression as compared to the non-metastatic CL1-0 cells. These results indicated that JARID1B is overexpressed in human NSCLC tissues and cell lines. To investigate the potential role of other KDM5 family members in NSCLC, we explored the Oncomine database and used The Cancer Genome Atlas (TCGA) database for comparison. We demonstrated that as compared with normal lung tissues from the same individuals, overexpression of KDM5B and KDM5C was found in NSCLC patients while KDM5A was not (Additional file 2: Figure S1).

### JARID1B overexpression correlates with advanced disease and poor prognosis

**Fig. 2:**
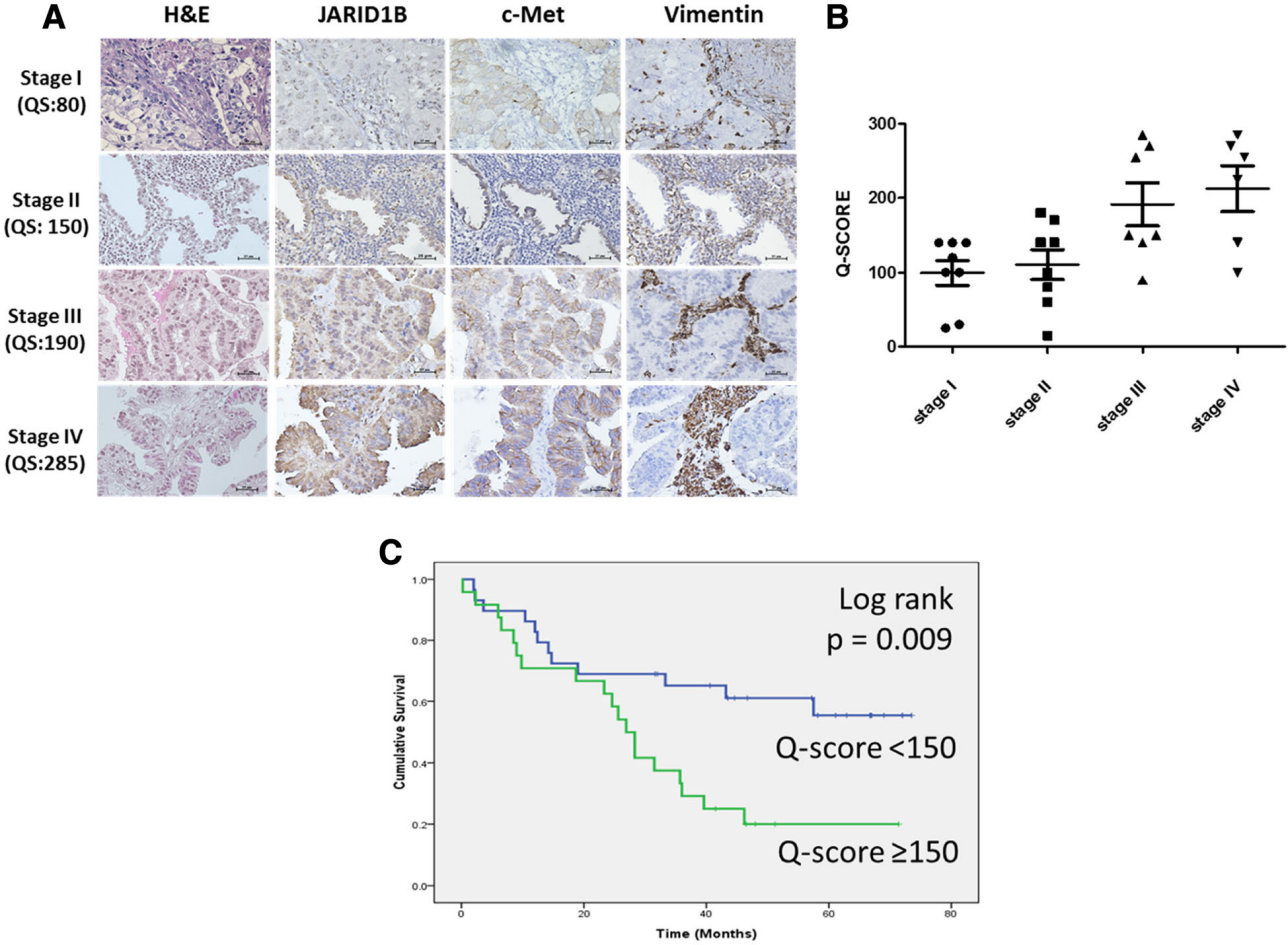
JARID1B overexpression correlates with advanced stages and worse overall survival (OS) in NSCLC patients. **a** Representative IHC staining of tissues from all-stage NSCLC patients showed concurrently increased JARID1B, c-Met, and Vimentin expression in stage 3 and 4 tumors as compared to stage 1 and 2 tumors. **b** Distribution of 29 NSCLC tumors with different stages according to Q-score values (*p* = 0.001). **c** The 5-year OS rates were 54.8% and 17.4% in JARID1B-low (*n* = 19) and JARID1B-high (*n* = 53) NSCLC patients, respectively (*p* = 0.009). Q-score ≥ 150 indicated JARID1B high and Q-score < 150 indicated JARID1B low

In the first cohort, we analyzed 29 patients of NSCLC with all stages. The representative pictures of each stage were shown in Fig. 2a. The IHC results showed that JARID1B overexpression was positively correlated with advanced tumor stages (*p* = 0.001) (Fig. 2b). Concurrently, a positive correlation was observed between the stage-dependent increase in JARID1B protein expression and enhanced expression of the tyrosine kinase c-Met and the type III intermediate filament protein Vimentin (Fig. 2a), where the former is implicated in cell proliferation, scattering and survival, while the latter is associated with the mesenchymal phenotype. In the second cohort, 72 NSCLC patients with stage I to stage III disease undergoing curative surgery were enrolled for clinical outcome analysis. Our results showed that patients with higher JARID1B expression (Q-score ≥ 150, *n* = 53) had significantly worse overall survival than patients with lower JARID1B expression (Q-score < 150, *n* = 19) (*p* = 0.009) (Fig. 2c). In the JARID1B-high group, 5-year overall survival rate was 17.4% with the median survival of 31.62 months, whereas in the JARID1B-low group, 5-year overall survival rate was 54.8% with the median survival of 50.15 months. As shown in Table 1, a significantly positive correlation between JARID1B expression and tumor size (*p* = 0.007, χ^2^ = 7.25), lymph node (LN) metastasis (*p* = 0.005, χ^2^ = 7.827), and tumor stage (*p* = 0.033, χ^2^ = 4.527). No statistically significant correlation between JARID1B expression and patient age (*p* = 0.737, χ^2^ = 0.112) or tumor differentiation (*p* = 0.382, χ^2^ = 0.828) was observed. These data suggested the potential role of JARID1B as a marker of tumor progression and a useful predictor of clinical outcome in NSCLC.

**Table 1:** Correlation between JARID1B expression and clinicopathological parameter in NSCLC patients

Parameters	JARID1B-low expression (*n* = 19)	JARID1B-high expression (*n* = 53)	χ^2^ value	*p* value
Age (years)				
≦ 50	8 (42.1%)	20 (37.7%)	0.112	0.737
> 50	11 (57.9%)	33 (62.3%)		
Tumor size				
≦ 2 cm	12 (63.2%)	15 (28.3%)	7.250	*0.007
> 2 cm	7 (36.8%)	38 (71.7%)		
Differentiation				
Poor	6 (31.6%)	23 (43.4%)	0.828	0.382
Moderate	12 (63.2%)	28 (52.8%)		
Well	1 (5.2%)	2 (3.8%)		
LN metastasis				
Negative	16 (84.2%)	25 (47.2%)	7.827	*0.005
Positive	3 (15.8%)	28 (52.8%)		
Stage				
I, II	14 (73.7%)	24 (45.3%)	4.527	*0.033
III	5 (26.3%)	29 (54.7%)		

*Statistically significant values

### JARID1B knockdown inhibits NSCLC cell proliferation and colony formation, cell migration, and invasion

**Fig. 3:**
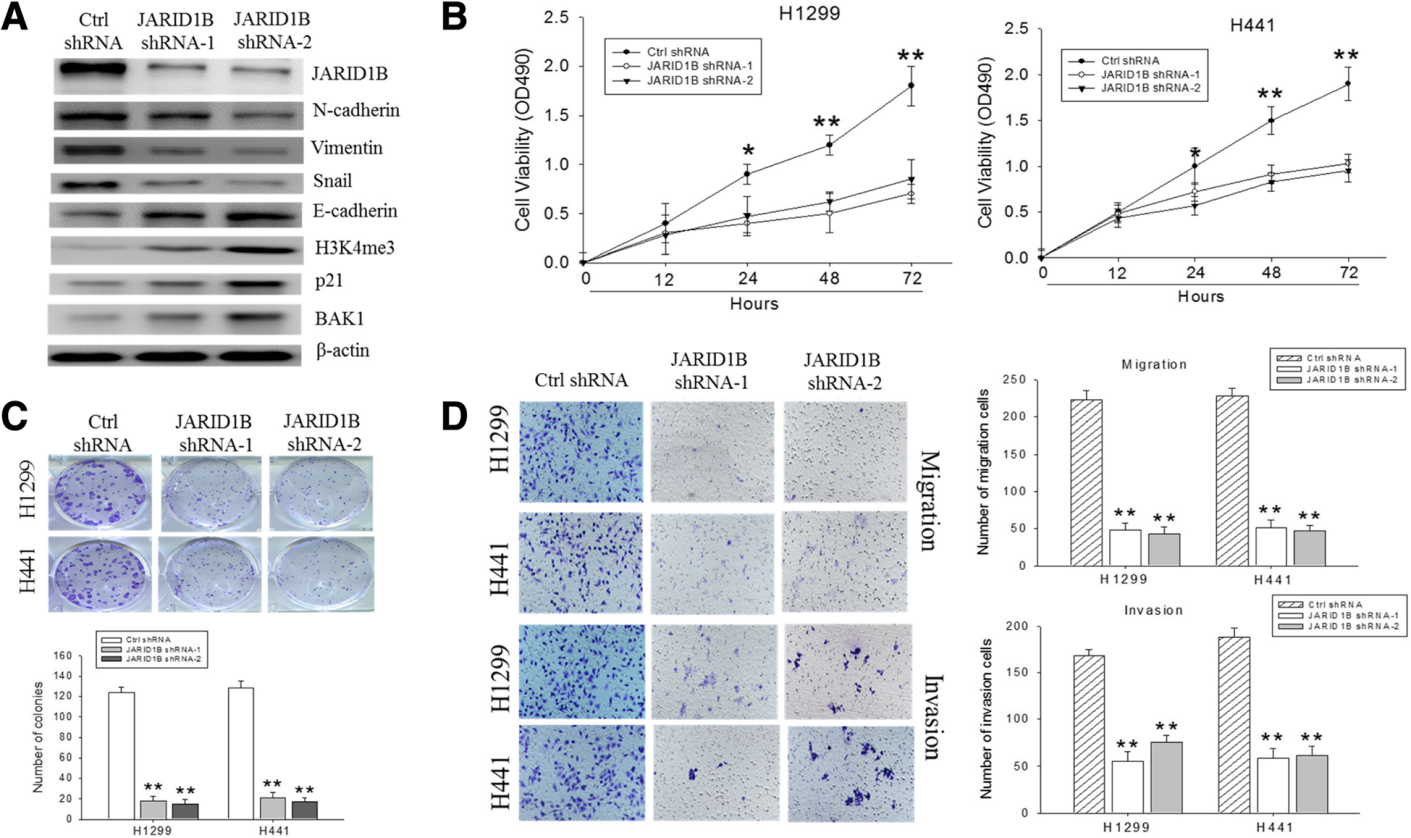
JARID1B knockdown changes EMT, apoptosis markers and suppresses cell proliferation, colony formation, and migration/invasion of NSCLC cells in vitro. **a** The knockdown efficiency of two JARID1B shRNAs (JARID1B shRAN-1 and shRNA-2) against endogenous JARID1B were evaluated by Western blot. Accompanied changes of several EMT markers and apoptosis makers were also noted. H3K4me3 increased after JARID1B suppression. ß-Actin served as the loading control. **b** SRB assay showed JARID1B knockdown suppressed cell proliferation. **c** (upper panel) JARID1B knockdown suppressed the ability of the H1299 and H441 cells to form colonies. (lower panel) Histograms showed significant inhibition of colony formation in the knockdown clones as compared to the control cells. **d** Staining of cells in migration assay and invasion assay (left panels) with crystal violet showed significantly reduced migration and invasion, respectively, in H1299 and H441 cells infected with JARID1B shRNA. (right panel) Histograms of the abovementioned data. The bars were representative of mean ± SEM independent experiments performed in triplicate assays. *p < 0.05; **p < 0.01. Original magnification, × 40

The cell line H1299 and H441 which expressed stronger JARID1B were used for knockdown study to determine whether JARID1B is necessary for cell proliferation and invasiveness of NSCLC cells. The JARID1B-knockdown efficiency in the shRNA-transfected H441 cells was verified using Western blot (Fig. 3a). The markers of epithelial-mesenchymal transition (EMT) were evaluated, and we found that the expression of EMT markers was parallel to the expression of JARID1B. The H3K4me3 activity and the expression of p21 and BAK1 were also increased after knocking down JARID1B, indicating not only enzymatic activity of JARID1B but also suppression of JARID1B may increase apoptosis. Consistent with this, result of our cell cycle analysis showed that depletion of JARID1B not only inhibited H441 cell proliferation via enhanced cell death, but also had an uncoupling effect on the NSCLC cell cycle progression as demonstrated by the shJARID1B-induced significant reduction in the population of cells in G0/G1 and S-phases, while increasing the number of cells in G2/M phase, which is indicative of reduced tumor cell growth and DNA replication, coupled with enhanced DNA damage (Additional file 3: Figure S3). Meanwhile, the SRB assay revealed that knockdown of JARID1B reduced cell proliferation remarkably in the H1299 and H441 cells (Fig. 3b). Reduced anchorage-independent growth in soft agar and lesser number of large colonies, as compared to the control groups, were also noted (Fig. 3c). Corresponding to the changes of EMT markers, significant inhibition of cell migration and invasion after 24 h was also observed in the JARID1B-knockdown cells in comparison to the control groups (Fig. 3d). Collectively, these data indicated that endogenous expression of JARID1B is essential for proliferation and formation of invasive phenotype in NSCLC cells, while both apoptosis and EMT phenomenon were important in these processes.

### JARID1B expression correlates with activation of the c-Met signaling pathway and facilitates CSC-like phenotype in NSCLC

**Fig. 4:**
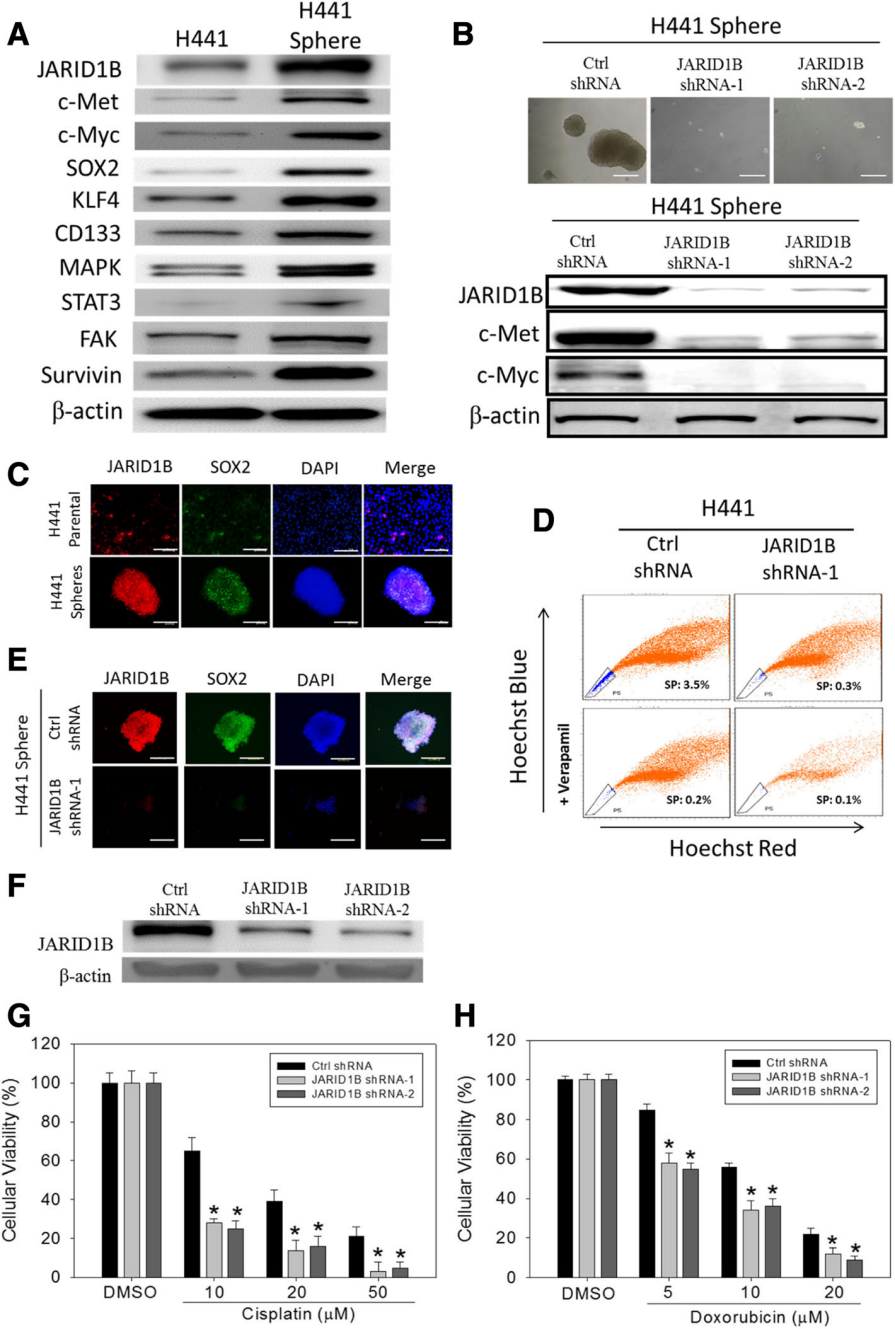
JARID1B expression activates the c-Met signaling pathway, facilitates CSCs-like phenotype in NSCLC, and JARID1B knockdown increases sensitivity to chemotherapy. **a** The expression of JARID1B, c-Met, c-Myc, SOX2, KLF4, MAPK, STAT3, FAK, survivin in H441, and H441 tumorspheres (spheroids) were shown. β-Actin served as the loading control. **b** Tumorsphere formation assay showed the inhibitory effect of JARID1B knockdown on tumorsphere formation (upper panel) as well as on the expression of c-Met and c-Myc (lower panel). **c** Immunofluorescence staining showing that H441 tumorspheres had greater expression of JARID1B and SOX2, compared with their H441-parental counterparts. **d** Fluorescence-activated cell sorter (FACS) analysis demonstrated the reduction of Hoechst 33342 efflux in shJARID1B cells as compared to the control H441 cells. The percentages indicated the proportion of side-population (SP) cells. The gated R5 region (blue) represented the Hoechst stain effluxing SP cells. The SP cell proportion reduced with verapamil treatment. **e** Dual-color immunofluorescence showed the co-expression and co-localization of JARID1B (red) and Sox2 (green). DAPI (blue) is the nuclear marker. Their expression was significantly diminished in the JARID1B-knockdown H441 cells. **f** Western blot for JARID1B in the control H441 cells and in the shJARID1B H441 cells. The control and JARID1B-knockdown cells were treated with increasing concentration of **g** cisplatin or **h** doxorubicin for 24 h. Cell viability was measured by SRB assay. *p < 0.05

To validate whether JARID1B expression is related to LCSCs, based on the documented evidence showing that markers such as c-Myc, OCT4, SOX2, KLF4, NANOG, and survivin are useful to define the LCSCs [8, 24], we evaluated the association between the expression of these markers and JARID1B by Western blot, immunofluorescent staining, tumorsphere formation, and flow cytometry side-population (SP) assays. Comparing JARID1B expression in H441 adherent cells and tumorspheres, we observed that JARID1B protein was expressed more in H441 tumorspheres as compared to the adherent cells, and this expression pattern was also noted for LCSC markers such as c-Myc, SOX2, KLF4, CD133, and survivin. Interestingly, c-Met and its downstream proteins including MAPK, STAT3, and FAK were also increased in H441 tumorspheres (Fig. 4a). This highlighted the possible involvement of the c-Met pathway between JARID1B and LCSCs. Additionally, JARID1B knockdown significantly diminished the ability of H441 cells to form tumorspheres, which were the in vitro models of CSCs, and correlated with significant downregulation of c-Myc and c-Met protein expression (Fig. 4b). Thus, the expression of stem cell markers JARID1B and SOX2 in wild-type parental and spheroid H441 cells were analyzed using the dual-color immunofluorescence staining technique. Results showed that the in vitro H441 tumorsphere models displayed significantly higher expression of JARID1B and SOX2, compared with their parental cell counterparts, H441-parental. Nuclear localization of these stem cell markers was also observed in H441 tumorspheres, as demonstrated by their immensely positive co-localization with DAPI staining (Fig. 4c). Furthermore, shRNA knockdown of JARID1B was sufficient to cause a 91.4% reduction in H441 side population (which exhibited higher efflux of DNA-binding dye Hoechst 33342 and were likely to be the LCSCs), an inhibitory effect comparable to what was obtained in verapamil-treated cells (94.3% in control H441 cells, 97.1% in shJARID1B H441 cells) (Fig. 4d). The immunofluorescent staining showed that while JARID1B and SOX2 were strongly co-expressed and co-localized in the nucleus of JARID1B-high H441 cells, their expression was significantly diminished in the JARID1B-knockdown H441 cells (Fig. 4e). Overall, these results documented the relationship between JARID1B and the LCSCs subpopulation and were suggestive of the active role of JARID1B in facilitating the formation of CSCs-like phenotype of NSCLC cells. Additionally, the c-Met signaling pathway was found to play a significant role in this process.

### JARID1B expression is associated with chemoresistance

We next evaluated whether JARID1B knockdown increases sensitivity to chemotherapeutic agents by depleting JARID1B expression in H441 cells with two JARID1B shRNAs (Fig. 4f) and measuring cell viability upon treatment of cisplatin and doxorubicin. Knockdown of JARID1B resulted in decreased cell viability as compared to the control vehicle (0.01% DMSO)-treated cells upon treatment with increasing concentrations of cisplatin and doxorubicin in H441 cells (Fig. 4g, h). These results suggested that JARID1B may contribute to chemoresistance in NSCLC cells.

### PBIT suppresses JARID1B expression and inhibits CSCs-like phenotype of NSCLC

**Fig. 5:**
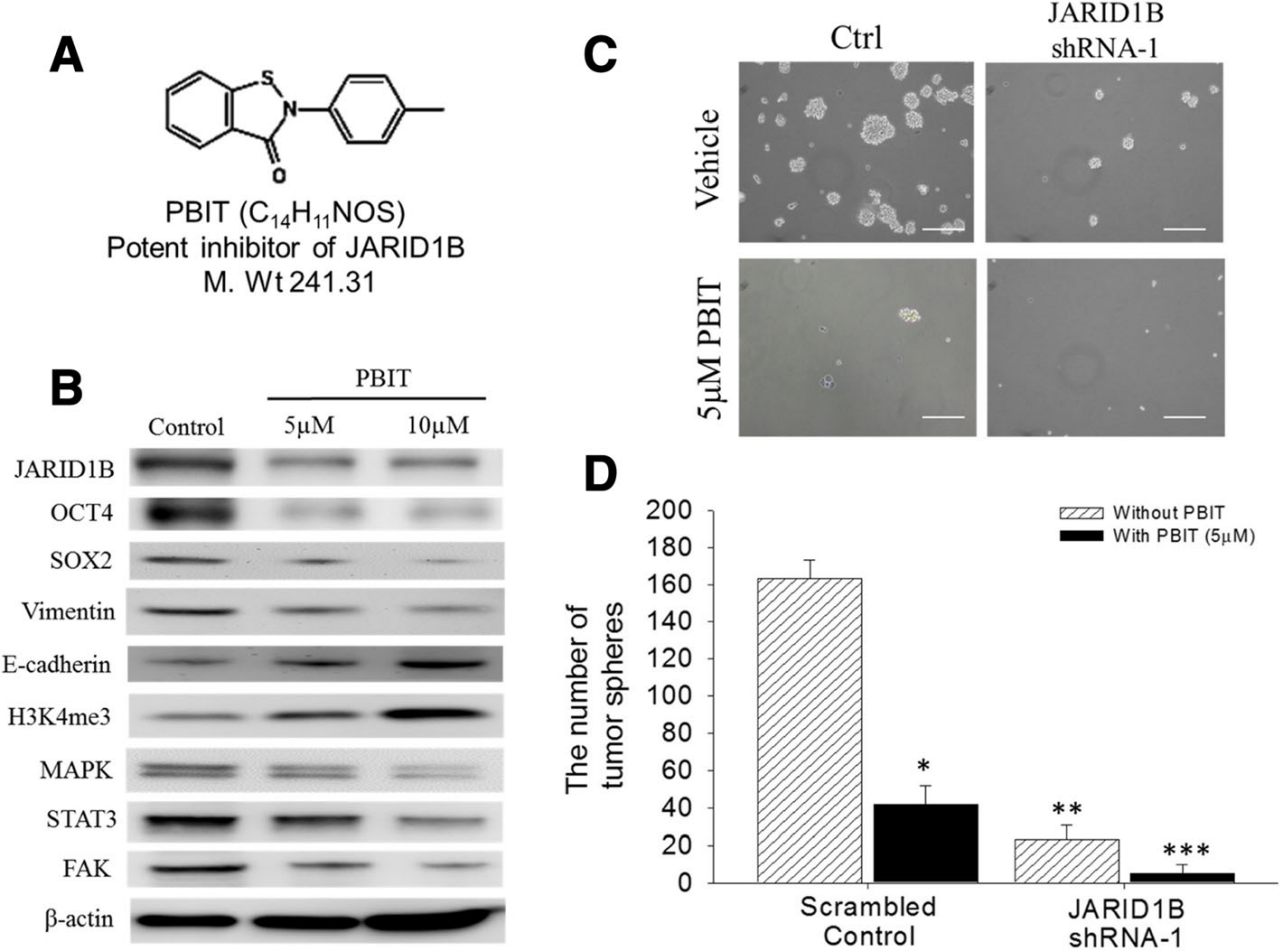
PBIT suppresses JARID1B expression and inhibits CSCs-like phenotype. **a** The molecular structure of PBIT (C_14_H_11_NOS), with molecular weight of 241.31 g/mol. **b** Western blot showed the dose-dependent inhibition of expression of JARID1B, Oct4, Sox2, vimentin, MAPK, STAT3, FAK expression, as well as upregulation of E-cadherin and H3K4me3 by PBIT treatment. β-Actin served as the loading control. **c** Tumorsphere formation assay showed the effects of JARID1B knockdown and/or 5 μM PBIT treatment in NSCLC-derived tumorspheres. **d** The histograms of data in **c**. shJARID1B, JARID1B shRNA-silenced; NC, negative control. The bars were representative of mean ± SEM of independent experiments performed in triplicate assays. *p < 0.05; **p < 0.01; ***p < 0.001

PBIT is a potent and specific inhibitor of JARID1B [25] (Fig. 5a), and we herein investigated the role of PBIT in NSCLC and evaluated its ability to suppress the potential and/or CSC phenotype of NSCLC cells. The Western blot assay demonstrated that PBIT significantly downregulates JARID1B protein expression in a dose-dependent manner. Similar to its inhibitory effect on JARID1B, PBIT also resulted in a correlative downregulation of OCT4, SOX2, Vimentin, and c-Met-associated proteins. At the same time, PBIT induced a dose-dependent upregulation of E-cadherin (Fig. 5b). Using the tumorsphere formation assay, we found that shJARID1B caused a significant (72.5%) reduction in the ability to form tumorspheres in NSCLC cells. In addition, 5 μM PBIT not only significantly suppressed tumorsphere formation (57.2%) in control H441 cells, but further potentiated the suppression of tumorsphere formation (95.6%) in shJARID1B H441 cells (Fig. 5c, d). These results suggested that PBIT suppresses JARID1B expression, diminishes EMT phenomenon, and inhibits the LCSCs phenotype, likely through the activation of the c-Met signaling pathway.

## Discussion

In the present study, we showed that JARID1B was significantly overexpressed in NSCLC tumor samples and cell lines, and JARID1B overexpression correlated positively with tumor size, lymph node metastasis, advanced tumor stages (Fig. 2b), and poor overall survival in NSCLC patients (Fig. 2c). The overexpression of JARID1B was associated with c-Met and Vimentin protein overexpression and was stage-dependent in nature. We then demonstrated that overexpression of JARID1B promoted cell proliferation, migration and invasion, drug resistance, and CSC-like phenotype of NSCLC cell in vitro. The cell line studies corroborated the clinicopathologic findings well and suggested that JARID1B may facilitate the oncogenic network of events through the epigenetic modulation of epithelial-mesenchymal transition (EMT) regulators including vimentin, snail, and E-cadherin, plus upregulation of a subset of pluripotent transcription factors such as OCT4, SOX2, KLF4, and c-Myc, and finally contribute to aggressiveness and stemness of NSCLC cells. Meanwhile, the c-Met signaling seemed to play a significant role in these processes. These results are consistent with previous studies implicating JARID1B as an oncogene and demonstrating the role of JARID1B to be a marker of disease progression and possible therapeutic target [18, 26–29].

There is accumulating evidence showing EMT in the malignant transformation of benign cells, endowment of increased motility, acquisition of an invasive phenotype, and subsequently disease progression [30]. EMT is principally characterized by the repression of the cell adhesion protein, E-cadherin, which inhibits the motility of malignant cells and inversely correlates with the upregulation of mesenchymal markers such as N-cadherin, vimentin, and snail, which are known suppressors of E-cadherin [30]. Consistent with these characteristics, our results indicated that JARID1B knockdown induced a corresponding downregulation of N-cadherin, vimentin, and snail, while conversely upregulated E-cadherin (Fig. 3a). We therefore hypothesized that expression of JARID1B and its alterations in the NSCLC cells are associated with loss of cellular polarity and cell-cell adhesion following diminished E-cadherin levels, detachment of malignant cells, increased cell motility, and finally metastatic dissemination. By demonstrating contrary events after JARID1B suppression, we established a positive correlation between JARID1B overexpression and enhanced NSCLC cell motility as well as acquisition of the EMT phenotype (Fig. 3d). We also showed that JARID1B knockdown resulted in upregulation of p21 and BAK1, therefore inhibited apoptosis and increased cell proliferation (Fig. 3a–c).

Based on accruing evidence coupling the induction of EMT with the acquisition of CSCs-like phenotype [31], plus the aforementioned results regarding JARID1B and EMT, we assumed that JARID1B could be both a mediator of NSCLC cell invasiveness and a modulator of its CSCs-like phenotype. Our Western blot results showed that overexpression of JARID1B was present in H441 tumorspheres and was associated with upregulation of pluripotency transcription factors (c-Myc, SOX2, KLF4), CSCs-facilitating oncogenic factor (c-Met), and the apoptotic inhibitor survivin (Fig. 4a). We further demonstrated that loss of ability to form tumorspheres, downregulation of c-Met and c-Myc proteins, loss of co-expression and nuclear co-localization of JARID1B with SOX2, as well as the significantly diminished proportion of side population, after knocking down JARID1B (Fig. 4b–e) or applying JARID1B inhibitor PBIT (Fig. 5). Taking together, we speculated that overexpression of JARID1B promotes NSCLC cell proliferation, cell motility, invasiveness, and tumorsphere formation in vitro, not only allude to a novel role of JARID1B in the modulation of EMT and CSCs-like phenotype in NSCLC cells, but also give a mechanistic insight into the clinical observations that NSCLC patients with stronger JARID1B expression are more prone to have metastasis and significantly shorter overall survival. In addition, pharmacological inhibition of JARID1B effectively suppressed these phenomena caused by JARID1B overexpression.

Our current results also revealed that JARID1B knockdown enhanced cell death induced by cisplatin and doxorubicin in NSCLC cells. We considered the underlying mechanisms to be complicated and possibly multifaceted. As previously reported, histone demethylases could either decrease [32] drug resistance in prostate cancer cells or increase [33, 34] drug resistance in several other cancer cells. As for the role of JARID1B, previous reports were few and most described that JARID1B overexpression increased drug resistance in melanoma in vitro and in vivo [35, 36]. One study including only human subjects suggested that JARID1B is associated with poor prognosis and chemotherapy resistance in epithelial ovarian cancer [37]. On the other hand, it is well known that drug resistance is one typical characteristic of CSCs [38, 39]. Whether our current finding is due to the biological effects of JARID1B itself or secondary to the CSCs phenotype resulting from JARID1B expression is still unknown and deserves further investigation.

## Conclusion

In summary, our study reveals JARID1B-mediated promotion of EMT and CSCs characteristics in NSCLC cells, shows that JARID1B can be a putative marker of tumor progression and poor prognosis in NSCLC patients, and suggests JARID1B may be a good molecular or pharmacological target in NSCLC.

## Additional files

Additional file 1: **Table S1.** Primary antibodies used (DOC 28 kb)

Additional file 2: **Figure S1.** Bioinformatics analysis using TCGA database. The comparative box plots between the normal tissue (left plot) and lung cancer tissues (right plot) indicate that a significantly higher expression level of KDM5B and KDM5C is detected in the NSCLC patients, with KDM5B being more prominent. **p < 0.01; ***p < 0.001, t-test. (DOCX 143 kb)

Additional file 3: **Figure S2.** The uncoupling effect of JARID1B on NSCLC cell cycle progression. (A) Depletion of JARID1B results in accumulation of G2/M cells by FACS analysis. H441 cells stably expressing Ctrl shRNA and JARID1B shRNA individually were stained with PI for the analysis of cell cycle distribution. (B) Quantification data for A. ** *p* < 0.01, t-test. (DOCX 153 kb)

## Abbreviations

CSCs: Cancer stem cells
FGFR: Fibroblast growth factor receptor
H&E: Hematoxylin and eosin
JARID1: Jumonji, AT-rich interactive domain 1
LCSCs: Lung cancer stem cells
PDGFR: Platelet-derived growth factor receptors
SRB: Sulforhodamine B
VEGFRs: Vascular endothelial growth factor
