# Supplementary figures and images for: Histone demethylase JARID1B/KDM5B promotes aggressiveness of non-small cell lung cancer and serves as a good prognostic predictor
Source: Clin Epigenetics. 2018 Aug 9;10:107. doi: 10.1186/s13148-018-0533-9 (PMC6085612; doi:10.1186/s13148-018-0533-9)

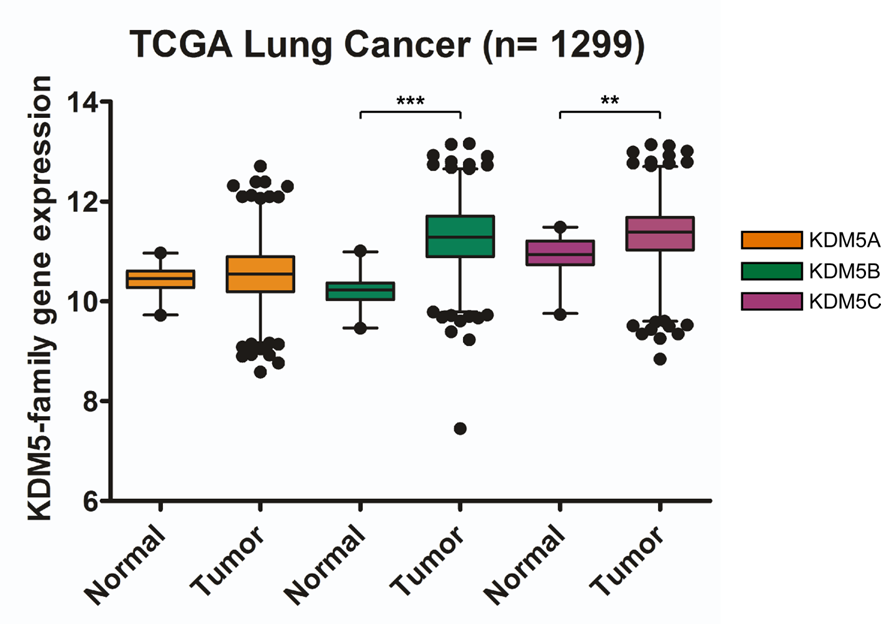


**Figure S1**

Supplement: Supplementary file 2 — Figure S1. Bioinformatics analysis using TCGA database. The comparative box plots between the normal tissue (left plot) and lung cancer tissues (right plot) indicate that a significantly higher expression level of KDM5B and KDM5C is detected in the NSCLC patients, with KDM5B being more prominent. **p < 0.01; ***p < 0.001, t-test. (DOCX 143 kb) [file 13148_2018_533_MOESM2_ESM.docx]

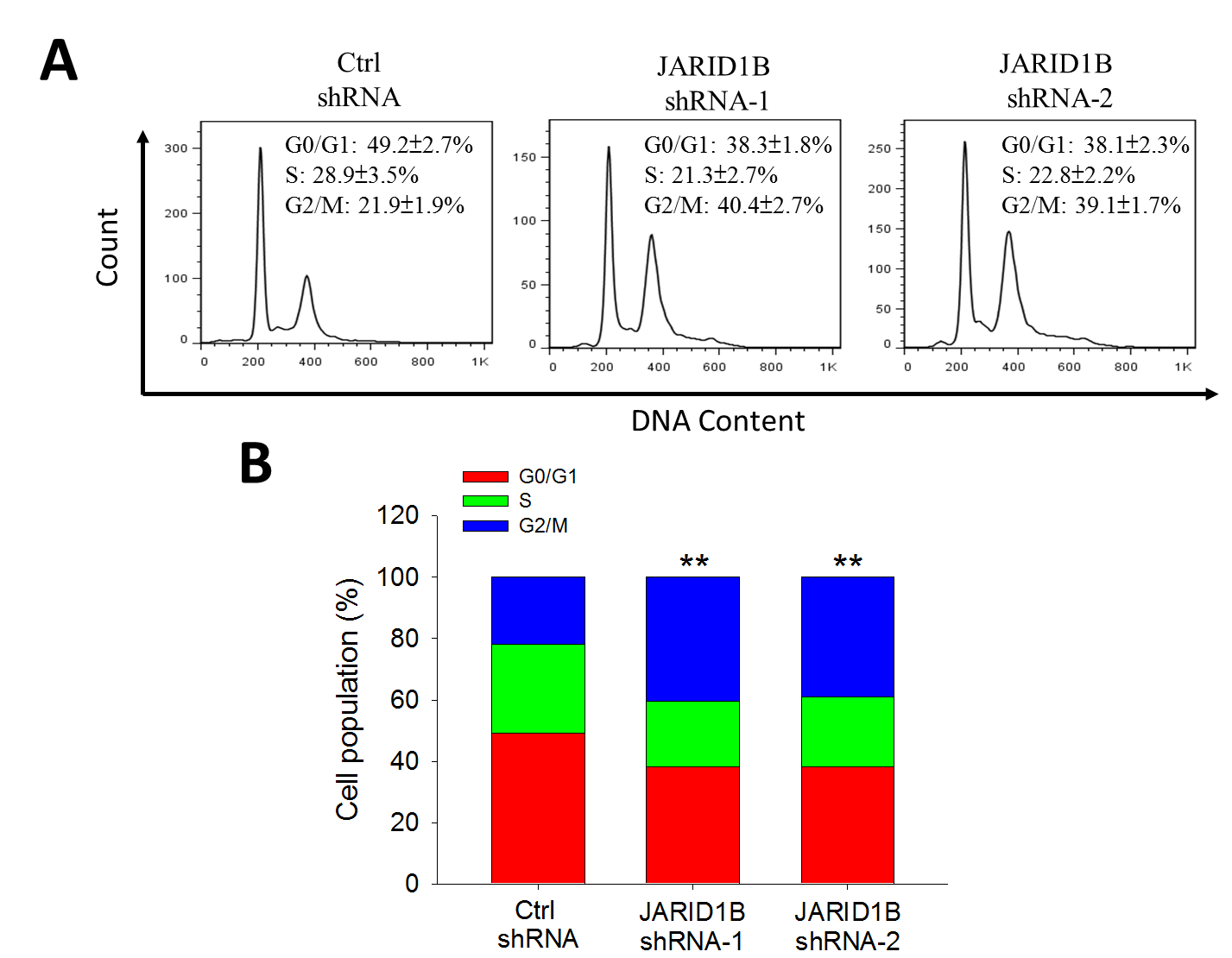


**Figure S2**

Supplement: Supplementary file 3 — Figure S2. The uncoupling effect of JARID1B on NSCLC cell cycle progression. (A) Depletion of JARID1B results in accumulation of G2/M cells by FACS analysis. H441 cells stably expressing Ctrl shRNA and JARID1B shRNA individually were stained with PI for the analysis of cell cycle distribution. (B) Quantification data for A. ** p < 0.01, t-test. (DOCX 153 kb) [file 13148_2018_533_MOESM3_ESM.docx]
