# Supplementary material for: Histone demethylase JARID1B/KDM5B promotes aggressiveness of non-small cell lung cancer and serves as a good prognostic predictor
Source: Clin Epigenetics. 2018 Aug 9;10:107. doi: 10.1186/s13148-018-0533-9 (PMC6085612; doi:10.1186/s13148-018-0533-9)
Supplement: Supplementary file 2 [file 13148_2018_533_MOESM1_ESM.doc]

Antibody	Species	Dilution			Compagny (Reference)
		IHC	IF	WB	
Anti-β-actin	Mouse			1:2000	Santa CRUZ (SC-47778)
Anti-Bak1	Rabbit			1:1000	Cell Signaling (#6947P)
Anti-CD133	Rabbit			1:2000	Proteintech (18495-1-AP)
Anti-c-Met	Rabbit			1:1000	Cell Signaling (#8198)
Anti-c-Myc	Rabbit			1:1000	Abcam (ab32072)
Anti-E-Cadherin	Rabbit			1:1000	Proteintech (20874-1)
Anti-FAK	Rabbit			1:1000	Cell Signaling (3285S)
Anti-GAPDH	Mouse			1:2000	Santa CRUZ (SC-47724)
Anti-H3K4me3	Mouse			1:1000	Abcam (ab1012)
Anti-JARID1B	Mouse	1:500	1:300	1:1000	Abnova (H00010765)
Anti-KLF4	Rabbit			1:1000	Cell Signaling (#4038P)
Anti-MAPK	Rabbit			1:1000	Cell Signaling (#9120)
Anti-N-Cadherin	Rabbit			1:1000	Cell Signaling (#4061)
Anti-P21	Rabbit			1:1000	Cell Signaling (#2947)
Anti-Snail	Rabbit			1:1000	Cell Signaling (#3879P)
Anti-Sox2	Rabbit		1:300	1:1000	Cell Signaling (#3579P)
Anti-STAT3	Rabbit			1:1000	Cell Signaling (#4904)
Anti-Survivin	Rabbit			1:1000	Santa CRUZ (SC-10811)
Anti-Vimentin	Rabbit			1:1000	Cell Signaling (#5741P)

The antibody dilutions are indicated for each application.
IHC, immunohistochemistry; IF, immunofluorescence; WB, Western blotting.
